# Exploring the barriers and facilitators to implementing electronic health records in a middle-income country: a qualitative study from South Africa

**DOI:** 10.3389/fdgth.2023.1207602

**Published:** 2023-08-04

**Authors:** Campion Zharima, Frances Griffiths, Jane Goudge

**Affiliations:** ^1^ Centre for Health Policy, Faculty of Health Sciences, University of Witwatersrand, Johannesburg, South Africa; ^2^ Medical School, University of Warwick, Warwick, United Kingdom

**Keywords:** electronic health records, implementation, barriers, facilitators, digital health information ecosystem, national health insurance, South Africa

## Abstract

**Introduction:**

As more countries are moving towards universal health care, middle-income countries in particular are trying to expand coverage, often using public funds. Electronic health records (EHR) are useful in monitoring patient outcomes, the performance of providers, and so the use of those public funds. With the multiple institutions or departments responsible for providing care to any individual, rather than a single record, an EHR is the interface through which to view data from a digital health information eco-system that draws on data from many different sources. South Africa plans to establish a National Health Insurance fund where EHRs will be essential for monitoring outcomes, and informing purchasing decisions. Despite various relevant policies and South Africa's relative wealth and digital capability, progress has been slow. In this paper, we explore the barriers and facilitators to implementing electronic health records in South Africa.

**Methods:**

In this qualitative study, we conducted in-depth interviews with participants including academics, staff at parastatals, managers in the private health sector, NGO managers and government staff at various levels.

**Results:**

The Western Cape provincial government over a 20-year period has managed to develop a digital health information ecosystem by drawing together existing data systems and building new systems. However, despite having the necessary policies in place and a number of stand-alone population level digital health information systems, several barriers still stand in the way of building national electronic health records and an efficient digital health ecosystem. These include a lack of national leadership and conflict, a failure to understand the scope of the task required to achieve scale up, insufficient numbers of technically skilled staff, failure to use the tender system to generate positive outcomes, and insufficient investment towards infrastructural needs such as hardware, software and connectivity.

**Conclusion:**

For South Africa to have an effective electronic health record, it is important to start by overcoming the barriers to interoperability, and to develop the necessary underlying digital health ecosystem. Like the Western Cape, provincial governments need to integrate and build on existing systems as their next steps forward.

## Introduction

More countries are moving towards universal health care, and middle-income countries in particular are trying to expand coverage, often using public funds. Digital interventions have considerable advantages for both patients and providers through increasing the efficiency of health service delivery and accessibility of health services (1). Electronic health records (EHR) enable monitoring patient outcomes and performance of providers, and so can improve the effective use of public funds (2, 3). The World Health Organization highlights that investment towards appropriate digital health solutions such as EHRs is important for low- and middle-income countries due to its potential to enhance delivery of healthcare, improve cost-effectiveness and enable the sustainability of health systems as well as delivering universal health coverage (4).

There has been a growing number of countries implementing electronic health records. By 2011, over 114 nations had initiated their implementation, including middle income countries such as Mexico, China, Brazil and India (5, 6). However, many of these efforts are still at an early stage primarily due to financial and technological challenges (7, 8). In 2005, the international community, led by the WHO, adopted long term resolutions on implementation of digital health information systems (9). Two of the resolutions were to create an enabling policy environment that supports digital health and encourages governments to invest in digital health solutions, and secondly, to develop strategies to guide implementation (4, 10).

In South Africa, in line with the global developments, a National electronic health (eHealth) Committee produced an initial 5-year strategy in 2010, although it was perceived to be too resource intensive to implement (11). A second South African policy, the 2012 eHealth strategy, was developed with a more consultative and iterative process to gather support from stakeholders (11). This was followed by the 2019 National Digital Health Strategy, which placed more emphasis on digital consumers and the potential of smart devices (4). Both strategies include the resolution to establish a complete electronic health record to effectively measure coverage and monitor service delivery and patient outcomes (12). They both propose the development of a roadmap to guide implementation and the integration of digital health information systems to a shared platform in order to advance functionality and continuity of care (12). In this paper, we report on the South African experience and explore the barriers and facilitators to implementation of electronic health records, to provide insights for EHR implementers in other middle income countries.

The burden of illness in South Africa, including HIV, TB, violence and injury, non-communicable diseases, and maternal, newborn and child health has a considerable impact on the health and wellbeing of citizens (13). Due to the two-tiered health system, which is divided along socioeconomic lines, there are significant disparities and inequitable access to health care (14). While South Africa allocates more resources towards health than the average of upper middle-income countries (15) and publicly funded health care in South Africa is available to all, its quality still needs improvement (16). Given the country's federal system of governance, the national department of health determines the policy direction, while the nine provinces are responsible for implementation. Recent policy initiatives envisage that a National Health Insurance (NHI) fund will be able to make better purchasing decisions through more formal contracting relationships (rather than through historical allocation of budgets by provinces), and so improve the quality of health services available to the majority (17). While progress towards NHI has been limited, as EHRs are key to monitoring the outcomes of care, they will be particularly important to assess the value obtained by the purchasing decisions of the fund.

## Methods

This study adopts a qualitative design and data was collected during the period from 05 November 2021 to 02 June 2022 with participants working in the field of digital health.

### Participants and sampling

Participant recruitment was initially done through purposive sampling and followed by snowballing through referrals from successfully recruited participants. This strategy was adopted in order to source data from specific people with relevant knowledge and experience. In selecting participants through referrals, we specifically asked participants for contacts whose views would differ from them in order to improve sample diversity and minimize bias.

All participants were invited to participate in the study via email, which contained a participant information sheet. Each participant was given a waiting period of one week to respond, after which, two follow up emails were sent a week apart.

### Data collection

Data was collected through qualitative in-depth interviews with consenting participants using a semi-structured interview guide as a data collection tool. The interview guide contained topics on the viability of the National Digital Health Strategy, existing digital health information systems in South Africa, the feasibility of implementing EHRs and the next necessary steps to prepare for the implementation of EHRs in South Africa. Due to COVID-19 restrictions and safety precautions during this period, 16 of the 18 interviews were conducted virtually using video conferencing software, either Microsoft Teams or Zoom. All interviews were conducted using the English language and each interview was audiotaped using an audio recording device.

### Data management

The data collected in this study was kept and stored in a password protected computer in the form of audio file recordings which were saved with generated unique participant identification numbers. We transcribed the audio files using the Otter transcribing software, checked the transcripts for accuracy and coded into themes using the NVivo software. To ensure participant anonymity, personal identifiers were removed from the transcripts and quotes used in reports were checked to make sure they do not reveal the identity of the participants.

### Data analysis

All interviews were coded by the lead author (CZ) and themes were identified using thematic analysis following the 6 steps outlined by Braun and Clarke (18). Using an inductive approach, the interview guide was used as a thematic analysis framework where broad topics were identified from interview questions on descriptions of existing digital health information systems, participants' thoughts on digital health policy landscape in South Africa, and factors affecting implementation of EHRs in South Africa. In addition to the list of health information systems provided, further information regarding the purpose, description, development and ownership of each system was supplemented by a separate internet search to provide a more complete picture of some of the existing systems in South Africa. The structure of the interview guide was formulated to explore the vision of digital health in South Africa as outlined by the National Digital Health Strategy, followed by a review of the progress and attempts made thus far with existing digital health information systems. Lastly, the guide explores the current barriers and facilitators to the implementation of EHRs in South Africa.

During data analysis a list of the names of all digital health information systems mentioned by participants was compiled and additional information on purpose, description, development and ownership was gathered separately through an internet search.

### Ethical approval

Ethics approval was obtained from the University of the Witwatersrand Medical Human Research Ethics Committee (M210213) as well as research permission from the National Department of Health, the Gauteng Provincial Department of Health (GP_202106_035) and the Health District research permission for Ekurhuleni and Johannesburg.

## Results

Data was collected from a total of 18 participants, mostly based in the Gauteng and Western Cape Provinces of South Africa. Participants were recruited from a range of professions which include: public health specialists from the district (*n* = 2), provincial (*n* = 3) and national (*n* = 3) office of the department of health (*n* = 8), staff from parastatals (*n* = 2), managers and staff from non-governmental organisations and non-profit Organisations (*n* = 4), academics in the field of digital health (*n* = 2) and managers in digital health private sector (*n* = 2).

In the following sections of our results, we describe firstly the policy landscape for EHRs in South Africa, the existing digital health information systems, the barriers and facilitators to implementation, and lastly our participants' views on the potential for EHRs in South Africa.

### The policy landscape

The National Digital Health Strategy received positive reviews from most participants: “*I think it [the digital health strategy] is a good policy. I don’t think there is anything wrong with the policy*”. **(Participant 3)** This support was partly based on the preparatory work. “*From what I've been involved in, there were consultations with people that should have been involved*”. **(Participant 14)**

Others saw the strategy as unfeasible: “*We punch above our weight as a country. We tend to compare ourselves with the developed world in terms of the policies that we put in place, without thinking about what is actually practical. So, we have all these grandiose idea without the necessary support and infrastructure to actually bring those policies into life*”. **(Participant 14)** Others argued that the policies set huge milestones for a 5-year period. “*I think they tend to overreach a bit, because in my experience, the reality is that it takes decades to really put foundations in and get things on the ground. And in a five-year plan you may get similar foundations in place, but you are not going to achieve everything in that time*”. **(Participant 2)**

While the strategy provides a high-level vision of the digital health priorities of South Africa, it does not have detailed guidance on how to move forward: “*The problem with a strategy is it’s just a loose and visionary document. It’s a great and necessary first step but to derive value from it you need to have a costed work plan with timelines so that people can move along a path to action. Otherwise, it just becomes a talking point and it doesn’t really drive any kind of action. The two need to go hand in glove; you need a strategy and then you need the costed roadmap with prioritized action points*”. **(Participant 1)** The provincial governments also need to work on tailoring the National Digital Health Strategy to their unique needs. “*It’s one thing to have a national strategy but if you want to move forward, you have provincialize it. Not one size shoe fits all*”. **(Participant 17)**

#### Health normative standards framework

One of the key drivers to the integration of health information systems has been the Health Normative Standards Framework (HNSF), which was jointly formulated by the Council for Scientific and Industrial Research (CSIR) and the South African national department of health to enable interoperability of digital health information systems across the country through adherence to its prescribed norms (19). The CSIR is a multidisciplinary research and development organization in South Africa which partners with the government's department of health among other stakeholders to provide technological expertise, consultancy and support services.

One participant describes how the HNSF was formulated “*It’s not something which we pulled it out of thin air, we looked at what is happening in other countries, with the help of the CSIR*”. **(Participant 17)** The HNSF thus sets standards for all health information systems including the private healthcare sector: “*There’s been quite a large push by vendors, obviously, to capture* [providing a service to] *more organizations because that is part of their business model, that the more records they have, the bigger their footprint. That has been nipped in the bud and the HNSF will prescribe what all health information systems in the country will have to adhere to*”. **(Participant 12)** The demands of the private sector vendors and their influence on politicians also needs to be managed: “The private guys are all trying to sell something, the vendors will be the ones we have to worry about. And the problem is they lobby the politicians. And we just have to be strong enough to say no. If the minister sends somebody to me or comes to me and says that this one said that I just say, no. We have a plan, and we are not being diverted from our plan”. **(Participant 16)**

However, despite the Government's stance on standards for interoperability, there have been engagements with the private health sector: “*The National Department of Health has been exceptionally careful in not alienating anyone or making it impossible to do business in South Africa, because we are trying to engage, and it is a national entity. They are trying to create jobs and to stimulate the economy, which is the overall objective of all departments*”. **(Participant 12)** The participant explained how this was achieved: “*they have engaged with the CSIR, which is a parastatal, and it is part of their mandate to facilitate the health normative standards and health information exchange as well as the information systems that would support it*”. **(Participant 12)**

Despite acknowledging the importance of the HNSF, one participant points out its shortfall. “*If you look at the health normative standards framework, we don't have all the standards that we need…*”. **(Participant 11)** Another participant outlines other challenges: “*The HNSF is not a very useful document in its current form; it’s actually just a shopping list of standards. So for me what’s missing is that middle bit where you have people who understand the architecture and can translate the thing and work closely with people to implement it*”. **(Participant 2)**

### Existing digital health information systems

Most participants said that there has been slow progress in moving towards an electronic health record: “There actually isn’t an electronic health record of any description in the public sector. Certainly, no unifying one. The systems in place are at best patient inventories and accounting systems”. **(Participant 5)** Another participant described the nature of most existing digital health information systems in South African public health care facilities: “They are more like back entry capture of paper registers. People are capturing information on paper and then giving it to a clerk to type it into a computer. That is not really an electronic health record, where the clinician has access to the digital record and is able to use that during consultation”. **(Participant 1)** In the private sector, the situation is different: “There is much a higher degree of electronic record and digitization. For example, you get some very sophisticated electronic health records in orthodontics. But there is a very low degree of integration [between providers]. They all stand alone, everyone’s fending for themselves”. **(Participant 5)** There is a degree of integration at the funder level: “because they [providers] are all required to submit stuff to funders. That’s why Discovery [a South African private health care insurer] has a fairly sophisticated analyses on large-scale public health trends”. **(Participant 5)**

One participant highlights the failed attempts by one province to implement an EHR: “*Gauteng attempted to do one of those [electronic health record] under an IBM-related system in the past and failed. They tried another one and that failed too. Even to this very day I’m not aware of them having an EHR implemented at Gauteng*”. **(Participant 5)** Similarly, in other provinces progress has been slow: “The North-West doesn’t have anything. They’ve got an accounting system and perhaps a patient inventory. And I don’t think anywhere, apart from the Western Cape, has got anything either. In other provinces where I’ve had some experience, there actually isn’t an electronic health record of any meaningful description. This is primarily because nobody can agree as to what the standards should be”. **(Participant 5)** However, provincial governments have partnered with private sector organizations to implement digital health information systems in health care facilities. One participant highlighted the challenge from their experience working with international firms to implement a health information system: “*It was a company from outside of the country, so they didn’t quite understand how the African system works, such as somebody waiting in a casualty for eight hours or … waiting eight months for a cataract operation. [The system] never really worked… although a lot of money was put into it and a lot of workshops were done*”. **(Participant 4)**

Participants named several digital health information systems that have been implemented in South Africa. From this information, a list of digital health information systems (Table 1) was compiled, and while this list is not exhaustive, it provides some context for our results. The systems listed include primary health care and hospital information systems. They range from large scale systems that are used nationally and provincially to smaller scale systems used at hospital or clinic level.

**Table 1:** Digital health information systems mentioned by participants that could provide data for an EHR in South Africa.

Type	Name	Purpose	Description	Development/ownership
Primary Health care level systems (National)	DHIS—District Health Information Software	To provide a tool for collection, validation, analysis, and presentation of aggregate and patient-based statistical data, tailored (but not limited) to integrated health information management activities.	District health information software is a national open-source health information platform. It moved to become a web based platform in order to make health data available as soon as it is generated.	The project was coordinated by the HISP (Health Information Systems Programme) Centre at the University of Oslo. It was originally developed to capture monthly data for districts in Cape Town in 1998 (20)
	Tier.net	To capture and report adequate data on HIV and TB care and treatment in public health facilities across South Africa.	A stand-alone patient inventory system with clinical information on HIV and TB management in health facilities. It digitizes the records of over 80% of the HIV population in the public health sector. Facilities combine data at district, provincial and national levels and report into the DHIS system.	A software development company (WAMTech) was retained to do the development of the TIER.Net software in collaboration with UCT. The information and data processed by Tier.net is owned by the National Department of Health (21)
	HPRS—Health Patient Registration system	To allow identity verification and record clinical visits in public primary health care facilities in South Africa.	The HPRS uses the South African identification number and other forms of legal identification to provide a Patient Registry and offers a Master Patient Index capability. Used nation-wide, it captures patient demographic details only and already has at least 44 million people registered.	This system is owned by the National Department of Health, through its contract with the Council for Scientific and Industrial Research (CSIR) to develop the HPRS (22).
	Electronic Vaccination Data System (EVDS)	To register, record, track and monitor Covid-19 vaccination progress in South Africa.	A national self-registration, online platform, built using the HPRS as a base code.	The information and data processed by the EVDS is owned and developed by the National Department of Health. The department works with a team of staff, health care workers and private service providers who interact with the data (23).
Primary Health care level systems (Provincial/District)	Primary Health Care Information System (PHCIS)	To centralize the registering of patients and record keeping by catering for entry of patient data, capturing of patient assessments and tracking of patient visits and outcomes.	A basic administration system that was integrated with other modules for Obstetrics and gynecology, booking and appointment as well as HIV and TB monitoring. It has been implemented in all public sector primary healthcare sites in the Western Cape except the City of Cape Town.	The system is owned by the Western Cape provincial government and was developed for public-sector community health sectors and clinics in the province. It provides demographic data for patient visits by using a unique patient identification number (administered by CLINICOM) that is attached to a patient's paper record as a bar code (24).
	E-tick	To record and store patient information regarding their visit to a facility. This information includes names, dates, illness and treatment and service provided at the facility.	An electronic/digital version of the paper tick register used to record patient data by health care providers and administrators. It has only been implemented in the Ekurhuleni district of the Gauteng province. The e-tick software is accessible using desktop computers or tablets.	The development and implementation of this system was funded by the Aurum Institute, an organization that partners with government, private sector and civil society for community health interventions.
Hospital Information Systems	Meditech	To document patient symptoms, diagnoses, comorbidities and medical history.	An electronic health record system which connects to other operational systems such as imaging, lab and pharmacy.	Meditech South Africa (Pty) Ltd is privately owned software which provides software solutions to healthcare organizations in South Africa (25)
	Medicom	To integrate data captured in Patient Registration, Appointments Scheduling, Out-patients Management, Trauma, In-patients Management, Medical Records and Patient Billing by health care professionals.	An integrated on-site electronic medical record system.	Privately owned by software vendors (26)
	Clinicom	To provide patient demographic and hospital administration data.	It is a system that supplies a unique patient identification number that is shared across other systems for public-sector users throughout the Western Cape. This system is now used in at least 52 hospitals across the province.	Privately owned by software vendors.This is used by nearly all hospitals in the Western Cape, providing patient demographic and hospital administration data (24, 27)

#### National and provincial systems

Table 1 shows health information systems by scale such as national, provincial, district and hospital information system. The District Health Information System (DHIS), Tier.net, Health Patient Registration System and the EVDS are core national systems used across South Africa. The National Department of Health has been involved in the development, implementation and ownership of numerous digital health information systems through collaboration with other stakeholders: “*Currently, we [Department of Health] are running I think twelve systems nationally under CSIR*”. **(Participant 6)** There has been an attempt at integration especially for systems that have a shared purpose, although the envisaged solution seems to be merging two systems in to one, rather creating an eco-system where different systems can speak to one another: “*The problem is that in the migration of data from the TB electronic register to TIER, there’s been many challenges which are yet to be resolved. One is the capacity at Provincial and District level, and that National has not put in place a sufficient number of people to do the troubleshooting*”. **(Participant 3)** Some participants mentioned the potential to build on the success of the Electronic Vaccination Data System (EVDS) developed in response to the COVID pandemic: “*The biggest single thing in health records nationally is something no one was even thinking of eighteen months ago, and that is EVDS, which currently has I think sixteen million people registered. That data just didn't exist before*”. **(Participant 8)** Another participant highlights one of the reasons for its success: “*There was support throughout. Firstly, they got the change management process right. We received one training from one source. I think that was the starting point that I feel was correct. The training management, they’ve done it perfectly*”. **(Participant 6)**

#### The Western Cape provincial electronic health record ecosystem

The Western Cape Province has been able to build a coordinated digital health ecosystem. Initially, the province implemented a hospital information system: “*The primary one was the Clinicom System which has a patient master index and was implemented in all hospitals. We began to use it as the Primary Index for the Province, it was largely administrative but with little clinical data. Linked to that is the Pharmacy System which looked after stock management and dispensing, and it now runs in all hospitals, most community health centers and some clinics. There was a billing system that was also linked to Clinicom*”. **(Participant 2)** In addition to this, the provincial government further implemented a primary health care system: “*We [Western Cape] implemented the primary healthcare system that we wrote ourselves which collects clinical information for visits [patient-clinician consultations] and does appointment scheduling and reporting. Then we did other systems, a forensic, a mortuary system and emergency medical and a picture archiving and communication system and radiology information system [PACS/RIS] which were all linked*”. **(Participant 2)** This enabled interoperability, sharing of health information across facilities and platforms in the province: “*So there is one coherent provincial health record that links the clinical record to national lab results to reporting into [District Health Information Software] DHIS2, reporting to donors, to specific campaigns like HIV and non-communicable diseases*”. **(Participant 8)** As a result of this work, the province can track patients and monitor large scale public health issues: “*We are reaping the benefits because these systems are part of the twenty plus systems that are pulled into the provincial health data center. We can link the data on individual patients and come up with a longitudinal health record. They can do disease cascades and infer disease or condition episodes*”. **(Participant 2)** The success of the Western Cape owed much to the steps that were taken by the provincial government to build a digital health information ecosystem, making progress faster than other provinces as one participant points out: “*Out of 55 hospitals in the Western Cape, 54 of them have electronic health records. In Gauteng we have 37 hospitals, but only two hospitals have them. We have about 20 plus community health centers [in Gauteng] and only two community health centers have implemented them*”. **(Participant 17)**

#### Hospital information systems

Participants mentioned hospital information systems which include Medicom, Meditech and Clinicom. Such hospital systems are often privately owned and developed to cater for the targeted needs of hospital operations. While the purpose and design of each system may depend on the needs of the health care facility, hospital systems generally have a transformative impact on clinician workflow as one participant points out: “*Back in the day we didn’t have such things as tablets and handwriting recognition or anything like that, so it was largely to capture an image rather than some sort of auto-recognition or the other horrible case of data capture by typing*”. **(Participant 5)** However, the implementation of hospital systems may have different impacts on different types of clinicians as one participant highlights: “*It is much easier for the pharmacists and the lab people who seem to be drawn to that much more readily. In radiology or imaging per se …. your metadata can be captured quite easily, with all the picture archiving systems these days*”. **(Participant 5)** However, the successful implementation of hospital systems requires the cooperation of the users: “*The greatest challenge is getting clinicians on board, who don’t relish writing notes and capturing information. They feel in one sense that they’re above that* … *You have to either insist or demonstrate the benefits of their information going in and what they get out, so that they don’t feel like they’re just data capturers*”. **(Participant 5)**

### Factors affecting EHR implementation

Participants identified critical factors for the implementation of EHRs in South Africa and brought attention to four main issues that need to be addressed namely; (a) leadership, (b) skills and expertise, (c) resources, funding and infrastructure, and (d) governance (see Table 2).

**Table 2:** Summary of factors affecting EHR implementation.

Factor	Details
Leadership	• Leaders that understand the scale of implementation, and obtaining buy in from various stakeholders for a collaborative implementation process. • Leadership appointments based on merit rather than political affiliations. • Internal conflicts leading to uncertainty and hindered progress.
Skills and expertise	• Capacity building to address skills shortage in the public health sector, collaborating with more skilled sectors can be helpful • Health care workers should be involved in the implementation process • Early training programs can prepare the health care workforce for implementation.
Resources, funding and infrastructure	• Long-term investments towards infrastructural needs (hardware, connectivity, etc.) • Increased funding for public health facilities in remote areas. • Setting up data storage centers to facilitate the integration and migration of health data. • Building on existing infrastructure while gradually upscaling.
Governance	• Alignment between provincial and national health departments for concerted implementation, transcending the federal system. • Better monitoring mechanisms for tender systems in to combat corruption and improve accountability. • Appropriately qualified and skilled authorities to prioritize digital health. • A transparent and multi-disciplinary panel of experts to help with planning and decision-making.

#### Leadership

Leadership is viewed as an important factor in facilitating implementation: “*You need strong leadership really and everybody to buy in because it’s in our collective interest*”*.* **(Participant 3)** One participant highlighted the role played by leaders when implementing at scale: “*I think the first barrier is understanding the scale and having high level buy in from the minister of health. I’m not saying that isn’t present, but I don’t think they have always understood the full project and the implications of taking on something like that if you’re talking about national implementation*”*.* **(Participant 1)** Having high level buy-in also improves long-term collaborations with stakeholders as one participant shared their experience of the Western Cape Province: “*It’s a matter of getting everyone on the same page. In the Western Cape I'd say the relationship between Provincial Government and University of Cape Town is pretty good, because we've had a long-standing collaboration and sharing of skills. [As a result], the Provincial Government had an openness to allow certain things, and I was able to deal directly with the Head of Department [Western Cape provincial government] and the Deputy Director Generals*”*.* **(Participant 2)** However, the lack of high level buy-in from organizational leaders in some cases led to a reduction in the available support to government: “*the decision was to get rid of Digital Health [unit] and that included my division and several others including Telemedicine [at the Medical Research Council]. So that was a real setback because after that there was a complete gap*”*.* **(Participant 2)**

Part of having good leadership requires putting the right people in place to lead and facilitate implementation. Participants criticized the appointment of leaders based on their membership to political parties at the expense of merit: “*I think the problem is [we] have people in positions of power that are there because [of their political connections], not because they were good logistics people or because they understood technology or anything else*”*.* **(Participant 9)** Others highlighted how the Western Cape Province has been able to make greater progress: “*The WC [Western Cape] Government does seem to a greater degree to get on with being a government as opposed to pushing party lines and deploying party cadres. And that’s probably why they’re a little further along; there is a sense of it is less about party politics and more about actually delivering as a government*”. **(Participant 5)**

Although the National Department developed the National Health Normative Standards framework to establish an overarching vision to integrate health information systems, internal conflict was noted: “*There is a lot of uncertainty in the Department about what belongs in the Department and what would belong in the NHI environment and in the NHI Unit. So, whether it’s information systems or electronic systems and the rest of it, that is all up it the air at the moment and because the Deputy Director General that was hired to do NHI is now the Acting Director General, and he has even less time and bandwidth to focus on anything other than the crisis the Department is currently facing*”. **(Participant 3)**

#### Skills and expertise

Participants argued that capacity building is an essential part of the process: “*Part of any comprehensive strategy to implement electronic health record needs to have a strong capacity building component*”*.* **(Participant 1)**, and that South Africa has people with the right skills to lead implementation: “*We have very knowledgeable people in the private information systems supplier domain. There are very knowledgeable people in the NGO domain. There are very knowledgeable people in the private healthcare sector, private hospitals, private funders*”*.* **(Participant 11)** However, other participants said that the skills necessary are in short supply in government: “*We don’t have skilled staff [in government] and we don’t have budgets to hire skilled staff*”*.* **(Participant 1)** Skilled people can be outsourced: ***“****We need to have a partner that we can work with to bring lots of IT skills, as a next best option, of course. The best option [though] is to have [the skills] in house*”*.* **(Participant 15)**

Participants noted the challenges when recruiting for the public health sector: “People are now reluctant to go work in the National Department for a number of reasons. One is they scared they going to be embroiled in controversy and their personal integrity might be at stake. The second is that the Department doesn’t pay as well as the private sector. So, attracting the right people is a big challenge and getting them to stay is also a big challenge”. **(Participant 3)** However, without sufficient skills, there may be limited progress: **“**If we do not bring in people that have the necessary skills to do the job, then we're always going to remain behind, as in other parts of the country”. **(Participant 14)** The National Department of Health needs to build a critical mass of people: “You need to create specific job categories for health information system workers inside the ministry of health and recruit people into those positions who have got some basic training and then send them on specialized training”. **(Participant 1)** A provincial health official added that the Department of Health needs to create job roles that involve clinicians: “Chief medical information officer is the way to go. You should have clinicians running information systems”. **(Participant 17)** One participant points out why this is important: “*So often the people that decide on systems are not the people that use the systems. So, the decisions are often taken by people … who do not understand the needs of the user. They go out and procure systems without realising that they're not solving the problems that the users had in the first place. And so, what you have is a system that’s not really useful to the user, but useful to you as a policy maker. So, they [users] won't support it, because it’s not useful to them, why should they implement it?*”. **(Participant 15)**

Participants highlighted the necessity of having a highly trained health care workforce for this vision: “So there must be a lot of training. Among the nursing staff computer literacy is really not that great. That needs to be fixed”. **(Participant 4)** Training needs to start early and accommodate everyone that joins the public health workforce: “..starting with training people at Universities. Pre-service is as important as in-service training. But training is an ongoing thing, I mean, it’s not a once off. It’s a continuous thing”. **(Participant 3)** The failure to train and prepare the workforce may lead to some problems as one participant pointed out: “*Our biggest challenge in the modern era is the human factor, in breaches of privacy through inadvertent sharing of data and access to systems, being hacked and being conned into security breaches. The systems are pretty much sewn-up with encryption, protection, back-up, data security, disaster recovery, but they’re only as strong as the weakest link, which is us humans*”. **(Participant 5)**

#### Resources, funding and infrastructure

Participants argued that digital health information systems require significant investments for infrastructure: “Our government is not doing enough in rolling out fibre. We rely on secondary infrastructure like Vodacom, or MTN [private sector companies] with whom now we have contractual issues”. **(Participant 6)** Much of the plans to develop a digital health eco-system are hampered poor connectivity: “we can't do what we need to do. Nearly 40% of public health facilities have no reliable connection to the internet. How do we run a real time data system that’s trying to run the national health insurance with portable health records?”. **(Participant 16)** Moreover, due to the currently inadequate rollout of infrastructure, there are geographical disparities: “In the remote clinics, it'll be a while before we get an integrated patient record that is operational”. **(Participant 16)** There is also insufficient resources to purchase both the hardware and the software: “*We need to source money for enablers. You can't think of buying and developing software systems if we know we're not going to have money for hardware… Often you have hardware, but you don't have system to load onto the hardware. And other times, you have software, but you don't have the hardware. [For example] there was a problem, and they ended up just giving computers to clinics, and by the time they return with software, the computer will be gone (stolen), because it was sitting in a box*”. **(Participant 15)**

As part of the infrastructural needs, one participant highlighted how the challenges related to the lack of storage facilities that are owned by the national department of health: “*We also have storage issues. Right now, as a department we are renting under Microsoft cloud*”*.* **(Participant 6)** Another participant further illustrates the importance of investing towards a data repository system towards the integration of systems through a central data storage centre: “*The other problem is that we have 10 departments of health, a national Department of Health, and then nine provinces, and they're all autonomous, but they don't have their own data centers, they all can run whatever they want to run. As a result, it’s really hard to do any kind of migration of data to a central data repository*”. **(Participant 9)**

One participant highlighted the importance of assessing what resources are needed in different areas of the country: “There must be a proper situational analysis done on what is available. It’s a massive job so maybe it would be good to cut it down to just the Metros first and then from the Metros do the big municipalities”. **(Participant 4)** It was suggested this should be followed by a costed work plan of the budgetary needs and a phased implementation would allow an adaptive learning experience: “You could take it [implementation] in stages. Definitely. You do well in one area, one local area and then you replicate that. By the time you start replicating it, you may have better ways of doing things”. **(Participant 2)**

Despite the need for more resources and better infrastructure, one participant argued that a lot can be achieved with the digital health information systems that are already in place: “*We do not need something that is expensive. I think EVDS proves us wrong, in terms of wanting fancy systems. We need something where we can scale it up when you add in modules. You do not have to add it up all at the same time, only gradually*”*.* **(Participant 6)** Another participant suggested that given the shortage of resources: “*the first thing to do is to actually see if you can utilize your existing resources [infrastructure], and only add more when you need them*”*.* **(Participant 13)**

#### Governance

Due to South Africa's federal system of governance, the national department of health has limited influence over provincial decisions, but: “*making sure that there is sufficient alignment of vision between national and province is absolutely critical. Otherwise, it will just not work*”*.* **(Participant 15)** A former national health official explained further: “*We’ve tried centralisation for setting norms and standards but if the money is not in the same place when you are setting norms and standards, nobody listens to you*”*.* **(Participant 3)**

Purchasing is done through a tender system, and one provincial health official pointed out how the tender system is often loosely followed and not monitored by responsible officials: “*The tender system is open to abuse. Who monitors whether a deadline is met? What is the consequence (of not meeting a deadline)?*”. **(Participant 4)** A poor tender system can lead to a negative reputation on the department of health: ***“****As a consequence [of corruption], the perception is that you can’t trust the National Department any longer. No one is going to give the National Department a big pot of money to run a tender until it can show that it’s got the systems and people in place who can do this thing without losing the money*”*.* **(Participant 3)**

However, one participant complained that the tender system didn't identify the right people to work with the department of health: ***“****The whole tendering system is not good. I've got a bunch of colleagues that I've worked with, over the last 20–30 years, that I trust, that I know that they can do what they say they're going to do. But the process doesn't allow me to even pick up the phone and speak to the guy. I have to go on a public website and get three quotes from arbitrary people that I have no relationship with. It’s like an arranged marriage*”*.* **(Participant 9)** While it is not appropriate to recruit one's colleagues, the process needs to ensure that skilled companies are willing to apply by providing technically adequate terms of reference, briefing sessions, and building confidence that the government will play its part appropriately, all of which require experienced people with skills and integrity in government: “*You can avoid it [corruption] if you have people with integrity running the system. If you have people of dubious levels of integrity, then you won’t necessarily get the best system. It starts there unfortunately. You’ve got to get people with integrity*”*.* **(Participant 3)** One participant argued that transparency and openness is important: “*There are established ways to procure and help one avoid the pitfalls of corruption, because it is a complex task. I would think it would be a good idea to set up a transparent panel of experts to assist with selection and with making those choices*”. **(Participant 1)**

### The potential for electronic health records in South Africa

Participants shared their thoughts on the potential for EHRs in South Africa, addressing the country's current position and the practical steps for advancement. One participant pointed out how South Africa is well positioned to make advances in digital health: “*Compared to the rest of Africa, South Africa is quite confusing. Clearly it has more resources, people, companies, NGOs and university involvement. However, that doesn't necessarily make things more coherent because there are many separate, often parallel, sometimes competing, different health information systems*”. **(Participant 8)** The history of how health care has been funded in South Africa increased the fragmentation: “*What happened in South Africa is that you had disease programs that had funding and they had their strategies for their own. And so, they started implementing digital solutions. And then they were driving those things. And there wasn't a strong body at the top saying hang on, we need to co-ordinate all of this in terms of the Digital Health Strategy*”. **(Participant 2)** One participant gave their account of how attempts at national leadership had failed: “*The implementation of the strategy should obviously be led by the NHISSA [National Health Information Systems Committee of South Africa] but it became weakened and there were these other programs that became stronger. There was fragmentation with power struggles within the Ministry. Despite the strategy being ambitious, certain things could have still been done if there were resources and the right people were in place to drive things through*”. **(Participant 2)**

Although relevant skills are available in the private health sector, participants described their concerns over the poor relationship between the National Department of Health and the private sector: “*In South Africa it has been particularly difficult for the public and private sector to partner for many reasons. The private sector tends to be focused more on billing systems and practice management systems that are focused more on their business model, where the public sector is focused more on public health, and to some extent the longitudinal patient record that is emerging now*”*.* **(Participant 1)** A former national department of health official attributes the poor relationship to a lack of trust between the public and the private sector: “*Relations are not good. They are awful. There are exceptions but I’m talking generally now. There’s no trust between the public and the private sector.*” However, collaboration is still necessary for progress to be made: “*There has to be a dialogue between policy makers, researchers, community members*”*.* **(Participant 3)**

One participant argued that there are two possible approaches to implementation: “*Many countries have implemented electronic health records without first having a strategy. Although it’s ideal if it’s implemented as part of a strategy, I don’t think it’s a necessary first step. So, some countries start top down, starting with a strategy and then implementation after while others start bottom up, starting with implementation then developing the strategy after. I’d say most countries follow the bottom-up approach*”. **(Participant 1)** The participant argued that most countries let different systems develop on the ground based on need, while ensuring interoperability, rather than trying to impose a single system from the top.

Other participants agreed, arguing that South Africa could move towards building a digital health ecosystem for EHRs by making use of existing systems: “*We can build off what EVDS has done. It’s limited in many respects, but it’s done the job in terms of getting patient level data and data by admission, gender, medicines and outcomes.*..*Take all that exists across all the hospitals, build on that and move on as long as it’s operable and the key word is inter-operability*…*I think they may have multiple entry points. You just have to agree nationally on what needs to be done and go for it*”. **(Participant 3)**

## Discussion

Our findings show that while South Africa has the necessary policies in place, little progress has been made to develop the necessary digital health ecosystem for a national electronic health record. Several e-health systems are currently deployed in different facilities across the country, but the integration of these stand-alone systems remains a challenge in most parts of the country. However, the Western Cape Province, over a 20-year period, was able to draw together data from existing data systems, and build new systems, which led to the creation of a provincial digital health eco-system and EHR. Much of this progress has been attributed to consistent and high-level political support and sufficient technically capable staff both inside and outside government. In contrast, other South African provinces are struggling with a lack of national leadership and conflict, a failure to understand the scope of the task required to achieve scale up, insufficient numbers of technically skilled staff, failure to use the tender system to generate positive outcomes, and insufficient investment towards infrastructural needs such as hardware, software and connectivity.

The WHO recommended that developing countries establish multi-stakeholder steering committees to help determine what is available in their country and undertake the suitable planning and development processes (28, 29). However, many middle-income countries like South Africa have also struggled with interoperability after implementing various digital health information systems.

India first published their national standards for EHR adoption, and then convened an expert committee established to support the adoption of EHRs (30). However, uptake of the EHRs was slow primarily due to the lack of universal patient identifiers, which affected the accurate coordination and integration across different systems. Similar to South Africa, India provides its citizens with free health care at government health facilities in order to address the challenge of financial vulnerability faced by over 40% of the country's population, as a step towards universal health coverage (30). EHRs in India are fragmented as the country still needs to address key issues such as improved infrastructure, a policy landscape, training for healthcare professionals and increasing partnerships between private and public health sectors (31). As reported in our findings, similar issues need to be addressed in South Africa for a successful EHR implementation.

China began by implementing regional EHRs with unique patient identifiers, followed by a series of guiding standards for interoperability (31). In its implementation, China followed Australia's 3-stage approach which begins with the piloting, followed by regional implementation and national EHR system, allowing both local and international vendors to provide solutions at different stages (32). Thus, with a universal patient identifier and interoperability standards in place, a regional and phased implementation of EHRs was the strategy used by China. However, nationwide interoperability is still poor despite the country's relative success with regional implementation of EHRs, and this is due to the lack of a national strategy for establishing standardized EHR systems until much later (30).

Brazil made notable progress in EHRs from as early as 2011 when the country initiated a national health card to establish a unified health record for citizens, followed by the provision of technical support and resources for EHR implementation in primary care facilities (30). As a result, different states and municipalities began using various information systems since the late 1990s, leading to challenges in integration to form a national electronic health record (33, 34). However, in 2019 Brazil implemented a National Health Data Network, a platform designed to enable the exchange of information across facilities in health care networks in both public and private sectors, creating a viable solution for their interoperability challenges.

In contrast, the lack of a proactive approach and commitment to dedicate resources towards the advancement of EHR adoption has slowed down South Africa's progress. Several studies show the importance of interoperability in addressing the fragmentation of digital health information systems (35–37). Given the current progress of the Western Cape Province in building a digital health ecosystem, other provinces in South Africa can identify the strategies used and challenges faced to draw insights that can guide implementation in other regions. To address the problem of fragmentation in South Africa, charting a path forward involves building on the existing health information systems. Widely used, population level systems such as the EVDS or the Health Patient Registration System (HPRS) are essential starting points for integration of systems (38). Out of the nine provinces in South Africa, five of them have some kind of operational EHR system in public hospitals (24), but some are from different vendors and built on different architecture, making it difficult to share information between healthcare facilities (39).

Participants highlighted the challenge of data storage and how the department of health needs a central data repository which is essential for the successful integration of health information systems. Some studies have shown that cloud based EHRs may offer a solution to data storage problems with lower costs, greater reliability of power supply, ability to facilitate much faster interoperability and greater analytical computing power (40–42). Concerns about data protection and cyber security can be met by ensuring that the servers providing the cloud-based services are located in a jurisdiction with at least the same, but preferably stronger, regulation of such issues.

It is evident that while South Africa has the necessary policies in place, the progress in implementing electronic health records has been limited. As described above, other middle- income countries have encountered challenges similar to what South Africa is facing. However, overcoming these challenges would require overcoming the barriers and drawing lessons from successful examples such as the Western Cape Province and from other middle-income countries and guiding the country towards a digital health ecosystem and an electronic health record system.

## Limitations

Due to the use of snowball sampling technique in this study, representativeness was not guaranteed as participants were nominating people they know and those who are also likely to share the same views with them. To mitigate this issue, we specifically asked for contacts of participants whose views would differ from other participants to improve sample diversity.

As data collection was conducted during a period of COVID-19 lockdowns, many health professionals were busy attending to the pandemic. As a result, some health professionals who may have had valuable insights could not participate in the study.

## Conclusion

South Africa plans to introduce the National Health Insurance to facilitate universal health care, and electronic health records are an integral part of this vision—to register and monitor patients who make use of the health system. However, developing a digital health ecosystem for an electronic health record requires long term commitment, adequate funding, critical skills and expertise and leadership that prioritizes and addresses digital health needs.

## Data Availability

The raw data supporting the conclusions of this article will be made available by the authors, without undue reservation.
